# Redox Active Antimicrobial Peptides in Controlling Growth of Microorganisms at Body Barriers

**DOI:** 10.3390/antiox10030446

**Published:** 2021-03-13

**Authors:** Piotr Brzoza, Urszula Godlewska, Arkadiusz Borek, Agnieszka Morytko, Aneta Zegar, Patrycja Kwiecinska, Brian A. Zabel, Artur Osyczka, Mateusz Kwitniewski, Joanna Cichy

**Affiliations:** 1 Department of Immunology, Faculty of Biochemistry, Biophysics and Biotechnology, Jagiellonian University, 30-387 Kraków, Poland; piotr.brzoza@doctoral.uj.edu.pl (P.B.); urszula.godlewska@interia.pl (U.G.); auzarowska@wp.pl (A.M.); annette.zegar@gmail.com (A.Z.); patrycja.kwiecinska@uj.edu.pl (P.K.); mateusz.kwitniewski@uj.edu.pl (M.K.); 2 Department of Molecular Biophysics, Faculty of Biochemistry, Biophysics and Biotechnology, Jagiellonian University, 30-387 Kraków, Poland; arkadiusz.borek@uj.edu.pl (A.B.); artur.osyczka@uj.edu.pl (A.O.); 3 Palo Alto Veterans Institute for Research, VA Palo Alto Health Care System, Palo Alto, CA 94304, USA; bazabel@stanford.edu

**Keywords:** antimicrobial peptides, defensin, chemerin, skin, gut, bacteria

## Abstract

Epithelia in the skin, gut and other environmentally exposed organs display a variety of mechanisms to control microbial communities and limit potential pathogenic microbial invasion. Naturally occurring antimicrobial proteins/peptides and their synthetic derivatives (here collectively referred to as AMPs) reinforce the antimicrobial barrier function of epithelial cells. Understanding how these AMPs are functionally regulated may be important for new therapeutic approaches to combat microbial infections. Some AMPs are subject to redox-dependent regulation. This review aims to: (i) explore cysteine-based redox active AMPs in skin and intestine; (ii) discuss casual links between various redox environments of these barrier tissues and the ability of AMPs to control cutaneous and intestinal microbes; (iii) highlight how bacteria, through intrinsic mechanisms, can influence the bactericidal potential of redox-sensitive AMPs.

## 1. Introduction

To execute a well-controlled response to microbial threats, body barriers largely rely on preformed and de novo synthesized molecules such as antimicrobial protein/peptides (AMPs), which can be functionally modified by microenvironmental cues. While the defining function of structurally diverse AMPs is to inhibit microbial growth, they often play a secondary role in host defense, for example as chemotactic and/or pro-inflammatory factors [1]. AMPs are variously distributed at epithelial surfaces and can be regulated post-translationally via several, often niche-specific mechanisms, including proteolysis and/or redox pathways. Whereas proteolytic modifications of AMPs are relatively well-explored, much less is known regarding how the diverse redox conditions present in different epithelial milieus can affect the ability of AMPs to shape microbial communities and prevent potential infection. Cysteine-based, redox active AMPs encompass many bactericidal factors that are crucial for skin and gut defense [2,3,4]. Here we focus on these redox-regulated AMPs and their role in protection against cutaneous and enteric microbes. We also discuss mechanisms underlying both host‑ and microbe-driven processes directed at modification of redox status and concomitantly the bactericidal potential of AMPs against bacteria and/or fungi.

## 2. Redox Sensitive AMPs in Skin and Intestine and Mechanisms of Their Redox-Regulated Activities against Microbes

AMPs are oligopeptides consisting of five to over one hundred amino acids that are commonly classified based on their signature secondary structures: α-helix, β-sheet, extended, and loop. Among these groups, α-helix and β-sheet structures are the most common [5]. Notably, peptide structures may change depending on environmental conditions. Peptides classified as α‑helical are often unstructured in aqueous solutions and undergo conformational shifts in non-polar environments [6,7]. This ability is attributed to an abundance of cationic and hydrophobic amino acids that comprise AMPs as well as sequence-intrinsic properties that permit formation of an amphipathic helical structure in a hydrophobic microenvironment [5]. Conformational change has been proposed to be critical for interactions with biological membranes. Certain larger AMPs, including defensins, display mixed structural components in their 3D structures [8,9], and are generally stabilized by intra-molecular disulfide bonds that restrict their conformations [10].

Direct interaction with the bacterial membrane is critical for the antimicrobial activities of most AMPs [11]. Both Gram-positive and Gram-negative bacterial cell membranes are rich in phospholipids like cardiolipin, phosphatidylserine and phosphatidyl glycerol, resulting in a net-negative charge. Likewise, lipopolysaccharide in the outer membrane of Gram-negative bacteria and teichoic acids in Gram-positive bacteria contribute to the overall negative charge of the bacterial cell surface [12]. Perhaps not surprisingly, most AMPs are positively charged, largely due to a high content of lysine and arginine residues, enabling electrostatic interactions of AMPs with microbial cell surfaces [13]. Besides absolute charge, the distribution of basic amino acids in AMPs is important for their biochemical and functional properties [14]. High hydrophobicity together with localized charge creates a tendency for peptide aggregation. However, when charged amino acids are distributed at both ends of the molecule, aggregation is less likely and peptide stability is increased [14]. Less commonly, AMPs are enriched in acidic amino residues, resulting in a net-negative charge. These peptides utilize different mechanisms, like binding metal ions, to interact with microbial membranes via salt bridges [15]. For most acidic AMPs, their specific mechanism of antimicrobial action remains unknown.

Several amino acids can be modified in a redox-dependent manner. Residues that mainly undergo oxidation in proteins and peptides are phenol and indole rings of tyrosine and tryptophan; sulfur-containing methionine; and cysteine residues [16]. However, even branched-chain amino-acids like valine undergo oxidation reactions [17].

We will focus first on the most well-characterized redox-dependent regulator of AMP activity: cysteine modifications. The end products of cysteine oxidation, aside from formation of disulfide bonds, are sulfenic (SOH) and sulfinic acids (SO_2_H) or *S*-nitrosylations [18,19]. Cysteine modifications that are fast and reversible are likely to affect antimicrobial properties of bactericidal molecules in epithelial barriers, including skin and gut‑associated AMPs (Table 1). Indeed, several AMPs have been reported to exist in situ both in cysteine-bridged and reduced forms, resulting in major conformational and functional changes affecting their antimicrobial functions [3,4,20].

**Table 1:** Skin‑ and gut-associated AMPs that are functionally modified by cysteine-dependent redox cues.

AMP	Gene	Expression Sites	Killing Mechanism	Oxidized Form	Reduced Form	Ref
hBD1	DEFB1	Keratinocytes; enterocytes, colonocytes (constitutive)	membrane lysis, forms oligomeric structure in reduced form	High activity against *E. coli* low against *B. adolescentis*, *B. breve*, *L. acidophilus*	High activity against *C. albicans*, *B. adolescentis*, *B. breve*, *L. acidophilus;*	[3,4,10]
hBD2	DEFB4A	Keratinocytes; enterocytes, colonocytes Skin; respiratory tract (all induced)	membrane lysis No known oligomerization-related activity	High activity, Gram-negative bacteria (i.e., *A. baumannii*, *P. aeruginosa*, *E. coli*, *K. pneumoniae*, *P. mirabilis*) Gram-positive bacteria (i.e., *E. faecalis*, *E. faecium*, *S. aureus*)	Low activity	[21]
hBD3	DEFB10 3B	Keratinocytes; enterocytes, colonocytes (all induced)	membrane lysis, inhibition of cell wall synthesis (target: lipid II)	High activity (*B. breve*);	No significant change in antimicrobial activity, diminished chemotactic activity	[8,10,22]
HD6	DEFA6	Paneth cells (small intestine) (constitutive)	reduced form: changes in bacterial cell envelope and disintegration of cytoplasmic structures, forms oligomeric structures	Low/no activity (against *E.coli*, *L. acidophilus*)	High activity (*B. adolescentis*, *B. breve*, *L. acidophilus*, *S. thermophilus*)	[3,23]
HNP1-3	DEFA1-DEFA3	Neutrophils, primary granules, (constitutive)	HNP1: membrane lysis, inhibition of cell wall synthesis (target: lipid II) HNP2: membrane disruption, aggregation and fusion of vesicles HNP3: membrane disruption, pore formation	High activity (*E. coli*, *S. salivarius*)	Low activity (*E. coli*, *S. salivarius*)	[23,24]
HNP4	DEFA4	Neutrophils, primary granules, constitutive	alters membrane permeabilization	Low activity (*B. adolescentis, L. acidophilus*)	High activity (*B. adolescentis*, *L. acidophilus*)	[24,25]
S100 calcium binding protein A7 (Psoriasin)	S100A7	Keratinocytes (constitutive at low level);proximal digestive tract (constitutive and induced)	membrane permeabilization (pH dependent), Zn^2+^ sequestration	High activity against *E.coli*; lower activity against *P. aeruginosa*, *S. aureus*, *S. epidermidis* and fungi (*T. rubrum*)	Broad spectrum antifungal activity (i.e., *T. rubrum*, *A. fumigatus*, *T. mentagrophytes*). High activity against *E.coli*	[2,20,26,27,28]
Chemerin (p4)	RARRES2	Keratinocytes (constitutive); liver; adipose tissue (constitutive)	rapid damage and degradation of cell membrane; targets bacterial electron transport chain	High activity (homodimers) (*E. coli, S. aureus*)	Low activity (monomer) (*E. coli*, *S. aureus*)	[29,30]

**Figure 1:**
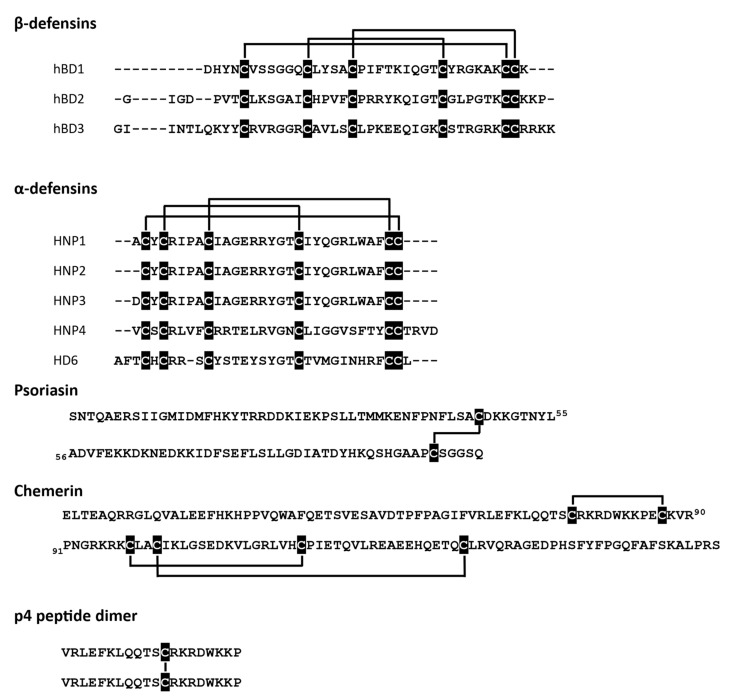
Redox-active AMPs with the indicated cysteine residues (black) and disulfide bridges.

At least three different antimicrobial mechanisms of redox-sensitive AMPs have been identified: (i) direct permeabilization of bacterial cell membrane, (ii) formation of oligomeric structures that can entangle bacteria, and (iii) ion sequestration that can lead to microbial cell death. The state of cysteine residues may define whether an AMP can effectively interact with a bacterial membrane. Certain AMPs show strong dependence on their ox-red form for microbial membrane interactions and cell entry. These include some skin- and gut-associated defensins, peptides characterized by six conserved cysteine residues that form three intramolecular disulfide bridges (Figure 1). Defensins come in two types, termed alpha (α)‑ and beta (β), based on the arrangement of their disulfide-bonds.

Alpha-defensins are mainly produced by neutrophils and Paneth cells in the small intestine at the base of intestinal glands, whereas β-defensins are produced by a variety of epithelial cells (Table 1).

Human β-defensin 2 (hBD2) or neutrophil-expressed α-defensins (HNP1-3) have been reported to be functionally modified by redox processes. HNPs show diminished antimicrobial potential in reduced form when compared to their native structures with intact cysteine bridges [23]. hBD2 is even more strongly dependent on redox modification, displaying a several-fold decrease in antimicrobial activity under reducing conditions [21]. Another AMP with three disulfide bridges, the chemoattractant and AMP chemerin may also be regulated by redox networks in barrier organs. Chemerin is expressed by epithelial cells, including skin keratinocytes [31]. Although chemerin antimicrobial isoform(s) in the skin remain to be determined, biochemical studies with chemerin-derived chemically synthesized peptides suggest that chemerin antimicrobial activity is primarily mediated by Val^66^-Pro^85^ fragment, peptide 4 (p4). P4 exhibits bactericidal properties against Gram-negative (*E. coli*) and Gram-positive (*S. aureus*) bacteria as well as fungi (*C. albicans*) [29,32] and can suppress bacterial growth in vivo when applied to the skin surface [30]. P4 is a redox active AMP capable of forming dimeric complexes in a cysteine-dependent manner (Figure 1) [30]. Both the monomeric form and the dimer interact with bacteria, but only the latter causes rapid damage and degradation of cell membranes. Moreover, components of the bacterial electron transport chain (ETC) have been identified as a target for p4 [30].

In contrast to hBD2, which is capable of restricting bacteria growth primarily when stabilized by disulfide bridges, another human defensin, hBD1, exhibits increased antimicrobial potential in a reduced, unstructured form. Both, redhBD1 and oxhBD1 can kill *E. coli* [10]. However, to unmask the antimicrobial activity of hBD1 against *Staphylococci*, *Lactobacilli* or *Bacteroides* species or fungus *C. albicans*, disulfide bonds in hBD1 must be reduced [4]. Taken together, these data suggest that the oxidation status of cysteines in β-defensins not only regulates their efficacy but also their target microbe specificity.

Similarly to hBD1, the antimicrobial activity of human defensin alpha 6 (HD6) has been experimentally linked to a reduction of the native oxidized form of HD6 produced by Paneth cells. While oxHD6 exerts little antibacterial activity against intestinal bacteria, including infectious microbes such as *Salmonella*, a reduced peptide shows increased potency against species like *Lactobacillus acidophilus* and *Bifidobacterium* spp. [3,23]. A truncated form of HD6, devoid of 2 amino-terminal residues, has been identified in vivo in ileum mucosa extracts and is more susceptible to alteration of its thiol-disulfide redox state and gains some antimicrobial potential under reducing conditions [23]. The functional modification of HD6 by both proteolysis and redox pathways highlights potentially more common mechanisms of regulation of AMPs that involve coexistence or co-dependence of peptide processing and modification of their redox status. Free sulfhydryl groups are not crucial for antimicrobial functions of redHD6, although reducing conditions are likely needed to generate an unstructured form of the peptide. This is suggested by the finding that substitution of cysteines with a steric, redox inactive cysteine analog α-amino butyric acid did not reduce but rather increased the antimicrobial potency of HD6 [23].

The redox-state of an AMP may also drive membrane-independent mechanisms of killing, like formation of oligomeric structures or ion sequestration. HD6 and hBD1 were shown to form oligomeric mesh-like structures that can effectively entrap bacteria, greatly limiting mobility and potentially spread of intestinal bacteria. hBD1 oligomerization is redox-dependent, requiring a reduced form of the AMP in order to create protein traps [3]. Generation of these structures involves cysteine residues, as hBD1 analogs with cysteine replaced with α‑amino butyric acid were unable to form net-like oligomers [3]. hBD1-derived traps were stable even in protease-rich conditions of human duodenal fluid and their assembly did not require microbial components [3]. While HD6 was first reported to form structures that can surround and entangle bacteria, including gastrointestinal pathogen-*Salmonella typhimurium,* via assembly into a net-like meshwork of fibril structures [33], the specific molecular mechanism likely differs from hBD1 since it does not involve cysteines. Instead, unique interaction of His^27^ and Leu^32^ in HD6 favors tetramer formation, leading to higher order oligomerization [33]. The initial oligomerization step appears to be triggered by stochastic, sequence-independent interactions with potential pathogenic microbes. However, without such a nucleation incident, HD6 remains in a mesh-free metastable form. Notably this initial nucleation step is much less likely to occur if the targeted protein is heavily glycosylated, indicating some preference towards microbial signals (which are in most cases aglycosylated) [33].

Antimicrobial mechanisms based on ion sequestration have been extensively studied for redox active S100A7 protein also known as psoriasin. Psoriasin is a 22-kDa homodimer expressed by epithelial cells, including skin keratinocytes [20,27]. Each subunit contains two cysteine residues that can be modified in a redox-dependent manner [26]. Psoriasin easily undergoes air oxidation. Therefore, oxidized psoriasin (oxPSOR) is the prevailing form found on healthy skin [2]. oxPSOR shows high antibacterial activity against *E. coli* [20,27], and, to a lesser extent, exhibits antifungal properties [2]. Importantly, the bactericidal activity of oxPSOR is pH-dependent. Permeabilization of cytoplasmic membranes occurs at pH below 6. This mechanism was observed primarily for Gram-positive bacterium *B. megaterium*. However, psoriasin does not form pores in the membrane of *E. coli* at either neutral or acidic pH [28]. Reduced psoriasin (redPSOR) exhibits high antibacterial (*E. coli*) and antifungal activity against filamentous fungi including dermatophyte *Trichophyton rubrum* and *Aspergillus fumigatus*, but not *C. albicans*. Zn^2+^ sequestration by psoriasin has been proposed as a redox dependent antimicrobial mechanism of action [2,20,27]. redPSOR penetrates the fungal cell membrane and induces apoptosis-like cell death as a result of Zn^2+^ sequestration from vital intracellular targets [2]. Antibacterial activity can also be mediated through nutrient deprivation in the extracellular space [26]. However, the chemical mechanism underlying the zinc chelating ability of human oxPSOR and redPSOR remains the subject of debate. Each subunit of psoriasin homodimer has two transition-metal-binding His_3_Asp sites. It was proposed that reduction of oxPSOR leads to the formation of additional thiol-based metal binding sites that may enhance Zn^2+^ sequestration [2]. On the other hand, another report demonstrated that the cysteine thiolates in redPSOR do not form high-affinity Zn^2+^ binding sites [26].

Redox conditions can also indirectly control AMP activity. RNase7 is a 14.5 kDa skin-expressed protein [34] that exhibits wide-spectrum antimicrobial activity against Gram-positive and Gram-negative bacteria as well as yeast [34]. The specific antimicrobial mechanism of action of RNase7 is unknown but is independent of RNAse7 nuclease activity [35]. The nuclease and antimicrobial activity of RNase7 is inhibited by RNase inhibitor (RI) [36]. Like RNase7, RI expression has been detected in human skin [37,38]. Only a reduced form of the inhibitor can bind RNase7. RI contains 32 cysteine residues that are reduced in its native form and can all undergo rapid oxidation once a single cysteine is modified [39]. Such changes cause RI to lose its ability to bind RNase, promotes RI degradation and, consequently, activates RNase7 [40].

Despite a high propensity for some AMPs to be redox-modified in a cysteine-dependent manner in vitro, it remains to be determined to what extent AMP cysteines are targeted for modification in vivo. The recent comprehensive and quantitative map of the mouse cysteine redox proteome in vivo (oximouse) indicate that highly modified cysteine sites are scarce [41]. Although skin and gut associated AMPs were not included in these studies, oximouse revealed that thiol redox sensitivity is likely encoded in an amino acid motif that depends on charged amino acids proximal to cysteine residues. The common amino acid signature that was found to be highly sensitive to redox modification involves selection for the positively charged amino acid arginine and selection against acidic amino acids (e.g., aspartic acid and glutamic acid). The presence of a positively charged arginine provided electrostatic stability for a negatively charged cysteine thiolate (which is much more sensitive to oxidation than a thiol), resulting in redox modification-permissive local biochemical feature [41].

Thus, functional modulation of AMP activity by redox-mediated processes is likely to be governed by two major variables; (i) the presence of a cysteine-containing motif in specific AMPs that is able to confer redox sensitivity to cysteine residues as well as (ii) specific tissue microenvironments capable of controlling cysteines already structurally poised to be modified by redox cues [41].

## 3. Redox Ecosystems of the Skin and Intestine in the Context of AMP Bactericidal Activity

Epithelial barriers offer a spectrum of redox properties, ranging from an overall pro-oxidative microenvironment at the skin surface and at the base of the crypts of the small intestinal, to a largely reductive environment at the lowermost layers of skin epidermis as and gut lumen. Consequently, the activity and functionality of redox sensitive AMPs is likely to be dictated by distinct metabolic and spatiotemporal redox-defining parameters across epithelial tissues.

***Skin*** is a barrier organ composed of three main compartments: epidermis, dermis and hypodermis. Epidermis, as the outermost layer of the skin, serves as the first line of defense against microbial assaults. Keratinocytes are the major cellular component of this layer. During epidermal differentiation, keratinocytes progress inside-out from mitotically active basal cells through spinous and granular cells of stratum spinosum and stratum granulosum to flattened, anucleated squamous cells (corneocytes) of the stratum corneum. Basement membrane separates epidermis from the underlying fibroblast-containing connective tissue, dermis. The lowermost skin compartment is hypodermis composed mostly of adipocytes [1]. All skin compartments produce AMPs as part of the host defense strategy [1].

Human and mouse skin are hypoxic, with baseline oxygen levels ranging from 1.5 to 5% [42,43]. Air oxygen diffusion into the skin is limited to the cornified envelope and outermost viable layers of epidermis, to a maximum depth 400 µm [44]. Since the epidermis has no vasculature, oxygen cannot be delivered by blood [45]. Epidermal germinal layers supply oxygen from papillary loops of dermal blood vessels [46]. Hypoxic conditions in the epidermis aid in pathogen clearance partly through stimulation of AMP production [47]. Differential oxygen accessibility in various skin environments may initiate or amplify host responses against cutaneous microbes not only by regulating expression levels of AMPs but also by mobilizing distinct bioenergetics pathways to control the activity of bactericidal molecules. These include modification of the redox status of cysteine residues in AMPs [2,4].

Given its location at the interface between body and environment, the epidermis is directly exposed to a highly pro-oxidative environment and oxidant assaults. In healthy skin, epidermal cells as well as fibroblasts produce reactive oxygen species (ROS) and reactive nitrogen species. Mitochondrial electron transport chain, nitric oxide synthase reaction and peroxisomal beta-oxidation are all important producers of endogenous free radicals [48]. Environmental assaults, including UVB radiation [49,50], xenobiotics [51], x-ray irradiation [52] and pathogens [53] are also either sources of ROS or stimulators of ROS production in the skin. To counterbalance pernicious skin-damaging oxidative stress conditions, skin is equipped with a variety of antioxidant systems. The outermost skin layer stratum corneum is rich in various antioxidants such as tripeptide glutathione (GSH), small proteins thioredoxin (TXR) and vitamins C and E [54]. In addition, keratinocytes as well as fibroblasts express antioxidant enzymes, including catalase or superoxide dismutase. The antioxidant capacity of epidermis is higher than dermis [55]. However, the activity of antioxidant enzymes is dependent on their location in the epidermis. This is exemplified by stratum corneum that exhibits decreasing levels of antioxidant enzymes activity closer to the skin surface [56]. The major ubiquitous factors implicated in maintaining proteins in their reduced state (low redox potential and high free SH levels) are reductase TRX and GSH. TRX is reduced by electrons from NADPH via TRX reductase, whereas GSH by NADPH and GSH reductase [57]. Both GSH and TRX are capable of conversion of psoriasin isoforms, such as oxPSOR and sulfitoPSOR (an isoform generated as a result of cleavage of disulfide-bridges by dermatophytes) into an antifungal agent in vitro, suggesting that they may also act in the skin environment as regulators of psoriasin activity [2]. The detection of both the Cys-oxidized and disulfide-reduced isoforms of psoriasin in the skin is consistent with in vivo regulation of psoriasin function by changes in its cysteine redox status. Levels of antifungal redPSOR in the skin increase during fungal skin infection [2], supporting the interdependence of cutaneous regulatory redox pathways with psoriasin-mediated antifungal responses.

High levels of free radicals coupled with lower activity of antioxidants contributes to increasingly oxidative conditions at the skin surface. Activities of many oxidants are higher in the epidermis than in dermis in both mouse and human skin [55]. AMPs produced in epidermis and operating at the skin surface can be expected to be modified by pro-oxidative conditions. These include antimicrobial chemerin peptides. Indeed, oxidative conditions substantially increase the efficacy of chemerin-derived peptide p4 to inhibit bacterial growth by facilitating formation of cysteine-dependent p4 dimers [30] (Figure 1).

***The intestinal epithelium*** is a complex and dynamic tissue composed of a single layer of different cell types. The small intestinal epithelium is extensively folded into crypts (containing proliferating stem cells) and villi (with terminally differentiated cells), while the colon epithelium lacks villi. The great majority of enteric epithelial cells are absorptive and highly polarized enterocytes. The remaining are secretory cells: mucus-secreting goblet cells, hormone-secreting enteroendocrine cells, and AMP-secreting Paneth cells [58]. Paneth cells are the only differentiated cells in the crypts, producing large amounts and varieties of AMPs. The most abundant antimicrobial peptides secreted by these cells are α‑defensins (HD5 and HD6), [59]. Furthermore, Paneth cell granules contain lysozymes, secretory phospholipase A2 (sPLA2), REG3α (belonging to C-type lectins), angiogenin 4, and cathelicidins [60].

The redox environment in rodent and human intestine is a highly reducing, with a redox potential of −150 mV in the ileum and −300 mV in the colon [61]. The reducing environment in the intestinal lumen is established by pools of glutathione/glutathione disulfide (GSH/GSSG), cysteine/cystine (Cys/CysSS) and thioredoxin/thioredoxin disulfide (TRX/TRXSS). Extracellular GSH levels in the intestinal lumen are very high (60−300 μM)—such large amounts of GSH derive from dietary intake and biliary output [62]. Luminal GSH participates in reduction of dietary disulfides, metabolism of peroxidized lipids, xenobiotic detoxification, and the assembly of mucin oligomers. The Cys/CysSS pool to a large extent contributes to maintaining the thiol-disulfide redox state of extracellular proteins [63]. The extracellular Cys and CySS concentrations are low (40 µM and 8–10 µM, respectively) and are constantly regulated by dietary Cys/CySS, GSH hydrolysis and thiol-disulfide exchange reactions [64].

One of the best functionally characterized redox proteins operating in the gut are intestinal TRX expressed in Paneth cells [65], acting in antioxidant defense and redox regulation through reduction of cysteine disulfides [66]. The redox potential of TRX1 and TRX2 is even lower than that of GSH/GSSG [67]. High levels of TRX are detected in intestinal epithelia [68], providing a physiological system that likely facilitates the antimicrobial functions of AMPs such as hBD1 against intestinal bacteria [69]. Ubiquitously expressed hBD1 exhibits low antimicrobial activity. However, upon contact with the reducing environment of the intestinal lumen, its disulfide bonds are likely reduced, for example by TRX or other lumen reductants. RedhBD1 displays greatly enhanced antimicrobial efficacy and potency as well as an ability to form “nanonets” that can potentially prevent bacterial translocation from gut lumen across the intestinal epithelium [4].

In vitro studies also show that reduction of HD6 can be catalyzed by TRX, but in contrast to hBD1, the antimicrobial activity of HD6 is less dependent on the generation of HD6 reduced isoforms. The potential functional consequences of HD6 reduction by TRX may involve maintaining homeostatic balance with colonizing microbiota in the small intestine, as redHD6 exhibits some bactericidal potential against non-pathogenic bacteria present in human intestinal microbiota (e.g., *Bifidobacterium adolescentis*) but not against pathogenic *Salmonella* [23]. As mentioned above, a key level of protection provided by HD6 is based on oligomerization and assembly into net-like structures that are cysteine-independent, and can entrap microbes, including *Salmonella*, limiting host-cell invasion by intestinal microorganisms [33].

Different dependencies of hBD1 and HD6 on redox regulation can be explained by differences in distribution of hBD1 and HD6 in the intestine, and variations in oxygen availability in the gut niches occupied by these AMPs. The partial pressure of oxygen (pO_2_) in the healthy colon is less than 10 mmHg, mainly due to the microbial biomass [70]. However, epithelial stem cells at the crypt base are highly oxygenated [experiencing a pO_2_ of ~100 mmHg] [71], a pressure similar to pO_2_ in healthy lung alveolus [72]. Such differences are a result of epithelial metabolism and the arrangement of the microvasculature network in each villous structure [71]. AMPs, like HD6, secreted by Paneth cells and constantly in transit towards the gut lumen, might operate in both the aerobic (oxygenated) environment found in small intestine crypts and the hypoxic and reducing milieu of the gut lumen [23].

In contrast, hBD1 secreted by different types of epithelial cells (including enterocytes and colonocytes) might be largely regulated by the reducing environment of the gut. Constitutive expression of hBD1 depends on basal HIF-1α signaling [73], and its oxidation is prevented by the low pO_2_ environment of the lumen [69].

## 4. Redox Pathways Involved in Reciprocal Interactions between Bacteria and AMPs

The importance of AMPs as endogenous bactericidal factors has generated interest in therapeutic approaches to combat antibiotic-resistant microbial pathogens with these antibiotic-like molecules. Of particular interest are AMPs that do not exclusively target processes that require high replication rates, since non-replicating or slow-growing bacteria can survive such approaches. Novel strategies may benefit from antimicrobial compounds, including AMPs, that interfere with the bioenergetics of pathogens [74]. Production of energy is necessary for all life functions, from sustaining growth to persisting in harsh environment (as exemplified by energy-dependent efflux pumps that can remove antibiotics from bacteria). Redox-active AMPs are likely to have an impact on key energy-converting pathways in microbes such as the respiratory chain that is driven by redox reactions. Moreover, the antimicrobial potential of AMPs might depend, at least in part, on their functional thiols and their ability to interfere with specific components of OXPHOS and/or reducing the magnitude of the proton-motive force (PMF) leading to the depletion of energy in targeted microbes [75].

All bacteria generate PMF across a proton-impermeable membrane. PMF is the difference in the concentrations of protons (ΔpH) and charges (ΔΨ) across a biological membrane. The formation of a proton gradient is coupled to the transport of electrons via membrane redox-active proteins (electron transport chain protein, ETC protein). These enzymes transfer electrons to each other via water-soluble protein (e.g., cytochrome *c*) or membrane non-protein (ubiquinone) electron and proton carrier molecules undergoing oxidation/reduction reactions.

PMF drives ATP synthase that couples proton transport with the formation of ATP from ADP and Pi. ATP is considered a universal “energy carrier” used by cells to carry out the biochemical reactions necessary for their survival. The metabolic pathway containing ETC along with the action of ATP synthase creates oxidative phosphorylation (OXPHOS, also called electron transport-linked phosphorylation). The proton gradient is also used for active transport of various compounds (substrates, metabolites) in and out of the cell. Therefore, it is extremely important to maintain PMF and membrane integrity.

Bacteria have developed different ways to generate PMF [74]. PMF can be generated by the action of proteins functionally similar to enzymes of the mitochondrial ETC: proton-pumping Complex I (NADH-ubiquinone oxidoreductase); proton-pumping Complex IV (cytochrome *c* oxidase); or proton-translocating Complex III (cytochrome *bc*_1_). Proton release may also be coupled to the operation of terminal reductase (for example, nitrate reductase). In some bacteria, efflux of the final product (e.g., lactate) may also generate PMF. Moreover, PMF can be generated when ATP synthase operates in a reverse direction, acting as an ATP-driven proton pump.

Considering all these factors necessary to build PMF, both membrane integrity and ETC proteins may provide novel targets for a new generation of antimicrobial compounds. These may include chemerin-derived p4, which is bactericidal against drug-resistant strains such as MRSA and can inhibit growth of either replicating microbes or to a lower extent bacteria in stationary phase [30,76].

Like most AMPs, high concentrations of p4 (above the minimal inhibitory concentration (MIC)), causes disruption of membrane integrity and bacterial cell death. However, below the MIC, p4 inhibited bacterial growth while having little effect on membrane integrity. Since the bacterial membrane harbors proteins involved in the ETC, we proposed that p4 may interfere with the function of ETC proteins. Indeed, p4 was a potent inhibitor of the enzymatic activity of cytochrome *bc*_1_ isolated from the purple bacterium *Rhodobacter capsulatus* [30]. Although it is likely that both bacteriostatic and bactericidal activity of p4 involves targeting the ETC components, it remains to be determined to what extent disruption of the membrane integrity is also dependent on p4 interference with the ETC.

Cytochrome *bc*_1_ is the central protein for various respiratory chains. It links the membrane ubiquinone pool and the cytochrome *c* intermembrane pool. Cytochrome *bc*_1_ protein participates in the building of PMF through proton translocation across the membrane coupled to reactions of oxidation/reduction of ubiquinol/ubiquinone. The interaction between cytochrome *bc*_1_ and its physiological partner, cytochrome *c*, is electrostatic in nature [77,78]. The surface of the binding domain in the cytochrome *bc*_1_ subunit, cytochrome *c*_1_, contains numerous acidic residues and is therefore negatively charged. On the other hand, the surface of cytochrome *c* interacting with cytochrome *c*_1_ is positively charged. Positively charged p4, and potentially other positively charged AMPs, can bind (through electrostatic interactions) to negatively charged cytochrome *c*_1_, thereby blocking access of the substrate, cytochrome *c*, to the active site. In this case, p4 acts as a competitive inhibitor for cytochrome *c* [30].

P4 is redox active due to a free cysteine. Oxidation of p4 leads to the formation of sulfide‑bridged peptide dimers that are equipped with bacteriostatic and bactericidal activity [30]. Among the potential oxidants of p4 in bacteria are high-potential redox-active cofactors of ETC proteins and/or ROS. For instance, in highly p4-sensitive Gram-negative bacteria, like *R. capsulatus*, the presence of functional cytochrome *bc*_1_ renders the cells highly susceptible to p4 action [30]. The reduction of cytochrome *c* by cytochrome *bc*_1_ is inhibited in the presence of oxidized but not reduced p4. Moreover, the free thiol group in the cysteine of redp4 was oxidized both in the presence of cytochrome *bc*_1_ and cytochrome *c* with formation of a disulfide bridge and dimerization of p4 (oxp4). The oxidation rate of p4 was higher in the presence of cytochrome *bc*_1_ than in the presence of cytochrome *c*. This may be a consequence of a higher redox potential (greater ability to accept an electron) of heme *c*_1_ of cytochrome *bc*_1_ than heme *c* of cytochrome *c*. Following this reaction, newly formed p4 dimers can function more effectively as antimicrobial factors [30]. Despite the fact that the cytochrome *bc*_1_ complex is missing in certain bacteria, including *E. coli* [79], other high-potential redox-active cofactors might cause oxidation of p4.

**Figure 2:**
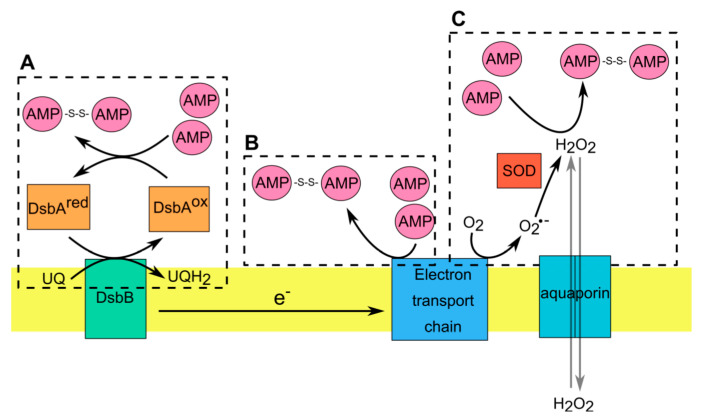
AMP interactions with the redox-active components in Gram-negative bacteria. (**A**). Disulfide bond formation is catalyzed in periplasm by DsbA (ox), that becomes reduced following the transfer of disulfide to substrate proteins (such as AMPs). DsbB mediates the reoxidation of DsbA via transfer of electrons from DsbA to the respiratory chain. (**B**). Within electron transport chain (ETC) some cofactors have a redox potential high enough to allow direct electron transfer from these cofactors to AMPs with formation of dimeric AMPs. (**C**). Hydrogen peroxide (H_2_O_2_) generated during normal or aberrant aerobic respiration induce the formation of disulfide bonds in AMPs. Microorganisms regulate the concentration of H_2_O_2_ via operation of SOD, which converts superoxide radical (O_2_^•−^) to H_2_O_2_ or aquaporins, which allow the transmembrane diffusion of H_2_O_2_.

In aerobic microorganisms, ROS are spontaneously formed during normal aerobic respiration and metabolism through the reduction of O_2_ by electron donors such as cytochromes, quinones and flavoproteins [80,81,82,83]. As a result of aberrant electron transfer in the respiratory chain, the formed superoxide (O_2_^−^) is detoxified by endogenous superoxide dismutases (SOD) to H_2_O_2_ [84]. Hydrogen peroxide is the most abundant and stable ROS that can easily pass through membranes [85]. Bacteria can regulate the concentration of H_2_O_2_ using aquaporins, which facilitate the bidirectional permeation of H_2_O_2_ across cellular membrane [86] (Figure 2). The presence of intracellular or extracellular H_2_O_2_ can modify the redox state of AMPs such as p4. For example, H_2_O_2_ induces the formation of disulfide bonds and subsequently increases the bactericidal activity of p4 in vitro [30]. Moreover, endogenous ROS generated by cytochrome *bc*_1_ in response to inhibition with antimycin significantly enhanced p4 activity [87]. Actively dividing bacteria in logarithmic (log) growth phase produce more O_2_^−^ than bacteria in stationary phase [83]. Therefore, if a logarithmic growth rate favors ROS generation, it may also sensitize bacteria to specific redox-active AMPs, such as p4. Recent studies have shown that both mitochondrial and bacterial cytochrome *bc*_1_ produce superoxide radicals in a rate dependent manner. The higher the activity of cytochrome *bc*_1_, the faster the rate of superoxide formation [88]. This is in line with increased ROS production and sensitivity to p4 in highly replicating bacteria requiring a high energy demand to sustain their growth [76]. However, it is also likely that other differences, such as an altered surface or cell membrane composition, may be responsible for the diverse effects of p4 on highly replicating bacteria compared to bacteria in stationary phase.

In Gram-negative bacteria there is an additional oxidizing system localized in the periplasm that can regulate redox-active thiols in AMPs. The envelope of Gram-negative bacteria is composed of an inner and outer membrane separated by the periplasm, which is absent in Gram-positive bacteria [80,89]. The periplasmic space is rich in soluble proteins that carry out important and diverse functions in bacteria such as protein folding, envelope assembly, ROS scavenging and nutrient import [80,89,90]. In contrast to cytoplasmic proteins, the majority of proteins in the periplasm contain oxidized cysteine residues. This is in line with a more highly oxidizing redox potential in the periplasm compared with cytoplasm [90,91]. In *E. coli*, disulfide bond formation in periplasmic proteins is catalyzed by two oxidoreductases: DsbA and DsbB [80,89,90,91]. Proteins that translocate to the periplasmic space react with highly oxidizing DsbA that acts as a donor of disulfide bonds. After introducing disulfide bonds into substrate proteins, the reduced DsbA needs to be reoxidized. This reaction is catalyzed by DsbB that acts as a quinone reductase, transferring electrons to ubiquinone or menaquinone under aerobic or anaerobic conditions, respectively [91]. Although the role of bacterial redox system DsbA/DsbB in oxidizing cysteine residues in AMPs remains obscure, recent evidence shows that both oxidoreductases contribute to controlling the activity of the oxidized form of hBD1 (oxhBD1) [92]. As mentioned earlier, oxhBD1 can control the growth of Gram-negative bacteria like *E. coli* but only in aerobic conditions [92]. The bactericidal properties of oxhBD1 are highly diminished in bacteria deprived of functional DsbA/DsbB, such as Gram-positive bacteria or *E. coli* mutants lacking DsbA/DsbB [92]. This mechanism of oxhBD1 bactericidal action requires two-step interactions with bacterial periplasm. First, oxhBD1 uses iron transporters like FepA and TonB to enter bacterial periplasm. Notably, TonB expression is repressed in anaerobic conditions, explaining this specificity. The second step relies on oxhBD1 interactions with bacterial oxidoreductases DsbA and DsbB, leading to hBD1 accumulation in the periplasmic space. This accumulation leads to bleb formation and cell lysis by a currently unknown mechanism [92].

Taken together, these data demonstrate that thiols in redox active AMPs can be reversibly oxidized following AMP interaction with respiratory chain components (e.g., selected ETC proteins) and products (such as ETC-produced ROS), or the periplasmic redox system of Gram-negative bacteria (Figure 2). Cell membrane and the periplasmic space are first points of contact between bacteria and AMPs and are strategically positioned to functionally tune interacting AMPs. AMPs can take advantage of the redox pathways in these cellular compartments to inhibit microbial growth.

## 5. Conclusions

**Figure 3:**
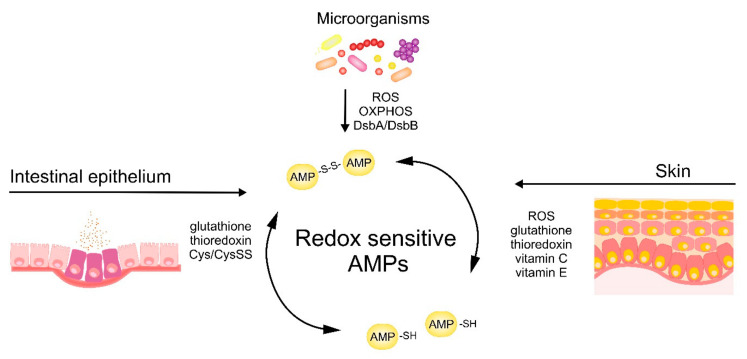
Skin‑, intestinal epithelium‑ and microbe-driven mechanisms influencing redox status and bactericidal potential of AMPs.

The antimicrobial activity of cysteine-based redox-sensitive AMPs is regulated by variety of environmental redox-mediated cues in skin and intestinal epithelium as well as redox-dependent processes in microorganisms (Figure 3). Understanding the molecular mechanisms governing the activity of AMPs at barrier surfaces will open up new avenues to effectively engage endogenous AMPs or deploy therapeutic synthetic AMPs to bolster epithelial immune defense.
